# Cost-utility analysis of valsartan, enalapril, and candesartan in patients with heart failure in Iran

**DOI:** 10.1186/s13561-023-00457-4

**Published:** 2023-09-04

**Authors:** Ramin Ravangard, Farideh Sadat Jalali, Marjan Hajahmadi, Abdosaleh Jafari

**Affiliations:** 1 Health Human Resources Research Centre, School of Health Management and Information Sciences, Shiraz University of Medical Sciences, Shiraz, Iran; 2 Department of Health Services Management, School of Health Management and Information Sciences, Shiraz University of Medical Sciences, Shiraz, Iran; 3 Student Research Committee, Shiraz University of Medical Sciences, Shiraz, Iran; 4 Cardiologist, Fellowship in Heart Failure and Cardiac Transplantation, Cardiovascular Department, Rasoul Akram General Hospital, Iran University of Medical Sciences, Tehran, Iran

**Keywords:** Valsartan, Enalapril, Candesartan, Markov model, Heart failure, Cost-utility analysis

## Abstract

**Background:**

Today, heart failure is one of the leading causes of death and disability in most developed and developing countries. By 2030, more than 23.3 million people are projected to die of cardiovascular diseases each year, and the prevalence of heart failure is expected to increase by 25%. One of the preventive interventions is pharmacological interventions which can be used to reduce the complications of cardiovascular diseases such as heart failure. One of the most important pharmacological interventions in patients with heart failure is the use of antihypertensive drugs such as candesartan, enalapril, and valsartan. This study aimed to compare the cost-utility of candesartan, enalapril, and valsartan in patients with heart failure using the Markov model in Iran in 2020.

**Methods:**

In the present study, a four-state Markov model was designed to compare the cost-utility of candesartan, enalapril, and valsartan for a hypothetical cohort of 10,000 heart failure patients older than 24 years. The payers’ perspective was used to calculate the costs. The Markov states included outpatients with heart failure, patients with heart failure admitted to general hospital wards, patients with heart failure admitted to the intensive care units (ICUs), and death. The effectiveness measure in this study was the quality-adjusted life years (QALYs). The one-way and probabilistic sensitivity analyses were used to determine the robustness of the results. The TreeAge Pro 2011 software was used for data analysis.

**Results:**

The results showed that the average expected costs and QALYs were 119645.45 USD and 16.15 for valsartan, 113,019.68 USD and 15.16 for enalapril, and 113,093.37 USD and 15.06 for candesartan, respectively. Candesartan was recognized as the dominated option. Because the calculated incremental cost-effectiveness ratio (ICER) value (6,692.69 USD) was less than the threshold value (7,256 USD), valsartan was cost-effective compared to enalapril. The results of the cost-effectiveness acceptability curve showed that at the threshold of 7,256 USD, valsartan had a 60% chance of being cost-effective compared to enalapril. The results of one-way and probabilistic sensitivity analyses confirmed the robustness of the results. Moreover, the results showed that ICU (1,112 USD) had the highest cost among cost items.

**Conclusion:**

According to the results, it is recommended that health policymakers consider the use of valsartan by cardiologists when designing clinical guidelines for the treatment of patients with heart failure.

**Supplementary Information:**

The online version contains supplementary material available at 10.1186/s13561-023-00457-4.

## Introduction

Heart failure (HF) is one of the leading causes of morbidity, mortality, and rehospitalization [1]. According to the American Heart Association, HF is a chronic and progressive disease in which the heart muscle is unable to pump enough blood to meet the body’s needs for blood and oxygen [2]. Symptoms of the disease and its complications over time cause limitations in the patients’ normal course of life so that the quality of life of patients with HF is lower than patients with other chronic diseases and they increase the risk of hospitalization and death [3, 4]. The American Heart Association (2014) reports that about 7.3% of all deaths from cardiovascular diseases are due to HF [5]. By 2030, it is predicted that more than 23.3 million people will die due to cardiovascular diseases annually, and the prevalence of HF is expected to increase by 25% [6].

Also, according to the results of a study conducted at the University of Utrecht in the Netherlands, about 64.3 million people worldwide live with HF [7]. According to other published statistics, the prevalence of this disease in the United States (2018) has been about 2.5% [8], in Germany (2013) over 4% [9], in the United Kingdom (2014) 1.6% [10], and in Belgium (2015) has been 1.3% in women and 1.2% in men [11]. The prevalence of HF in Iran in 2013 was reported to be 8%, which has been higher than that reported in other Asian countries, including Japan (0.8%), China (3.5%), and India (0.3%) [3, 7, 12]. While the incidence of HF is decreasing in developed countries, the prevalence is increasing due to the aging of the population, and the availability of effective treatment [13].

On the other hand, this disease is one of the common diseases that impose a great financial burden on individuals and communities [14]. The results of Harkness’s study (2015) showed that HF was related to high mortality, frequent hospitalizations, and a heavy financial burden on the health system [15]. Wu et al. (2013) have stated in their study that it is expected that by 2030, about 8 million Americans will suffer from HF, with costs related to their treatment amounting to 53 billion United States Dollars (USD) [16]. In Iran also about 23% of the burden of diseases has been related to cardiovascular diseases [17] and the annual cost of each patient with cardiovascular diseases has been reported as about $ 7,736.19 Purchasing Power Parity (PPP) [18]. Also, the rate of patients with HF in Iran is 3.3 per 100 people, and the hospitalization rate due to HF is about 0.3% per year. In recent years, the economic burden of HF in less developed countries has increased up to two times, and in Iran is about 400 billion rials (equal to 9,445,100 USD) per year [19, 20].

But the prevention of cardiovascular diseases, including HF, is applied at different levels. Preventive interventions and activities lead to a 20 to 30% reduction in the incidence of cardiovascular diseases and their mortalities as well as an increase in the quality of life [21]. One of the preventive interventions is pharmacological interventions which can be used to reduce the complications of cardiovascular diseases such as HF [22, 23]. One of the most important pharmacological interventions in patients with HF is the use of antihypertensive drugs such as candesartan and valsartan, which belong to a family of medicines called Angiotensin Receptor Blockers (ARBs) and enalapril (an angiotensin-converting enzyme (ACE) inhibitor). ACE reduces the angiotensin level. This decreases the overall peripheral resistance without increasing the oxygen demand of the heart [24]. Also, the binding of angiotensin to the receptors causes vasoconstriction and increased blood pressure. By blocking the angiotensin receptor, valsartan and candesartan dilates blood vessels and lowers blood pressure [25].

These medicines, with their three main mechanisms of nitric oxide release, potassium channel opening, and calcium channel occlusion, reduce vascular resistance and pressure and may reduce the risk of hospitalization for HF [26–28]. The safety and effectiveness of these three medicines in reducing mortality and morbidity have been investigated in various studies, and the use of these medicines is recommended by European and American medical guidelines [29–31]. However, although the benefits of these medicines have been proven for populations around the world, including Asian countries, it is necessary to obtain the necessary information to determine the most cost-effective medicines [32].

The cost-utility analysis is one of the most common forms of full economic evaluation studies in the health sector, which compare both costs and quality-adjusted life years (QALYs) of alternative interventions. These studies include both costs and outcomes. Outcomes are expressed as the incremental cost-effectiveness ratio (ICER), where differences in costs and outcomes are compared between two treatment options [33].

In this regard, some studies have been conducted in the world on the cost-utility of medicines related to HF. For example, a study by Ademi Zanfinaae et al. (2017) in Switzerland showed that valsartan, compared to enalapril, reduced the number of hospitalizations by about 6% per year and the lifetime hospital costs by 8% and improved the QALYs by 0.42. Also, the ICER for sacubitril/valsartan.

treatment versus ACE was CHF $25,684 per QALY gained [34]. In a study by Gazziano et al. (2016) in adult patients with HF in the United States, the results showed that the number of patients admitted to hospitals in people taking valsartan was 230 per 1,000 people less than those taking enalapril and, compared with enalapril, the strategy of using sacubitril/valsartan had an ICER of $45,017 per QALY gained [35]. Also, the results of a study conducted by Granstrom et al. in Sweden (2012) showed that candesartan, compared to losartan, had more effectiveness and QALYs and lower costs in patients with hypertension [27].

It is worth mentioning that despite the production of three medicines of candesartan, enalapril, and valsartan in Iran, an economic evaluation study on the cost-utility of these medicines was not found by researchers. Therefore, the present study was conducted to compare the cost-effectiveness of candesartan, enalapril, and valsartan in patients with HF from the payers’ perspective using the Markov model with the lifetime time horizon in Iran in 2020. In the current study, direct medical costs were considered and the effectiveness measure was the QALYs.

The results of this study can lead to determining the cost-effective medicine among the three medicines to be used by clinical specialists to control and treat the disease of patients with HF and also to be used in making decisions and designing clinical guidelines for proper planning for prevention and control of HF in the country.

## Methods

### Model design

To conduct the study, the four-state Markov model was used to compare the cost-utility of candesartan, enalapril, and valsartan for a hypothetical cohort of HF patients older than 24 years [36]. The model had been validated and used in the study conducted by van der Pol et al. in the Netherlands [37].

**Fig. 1:**
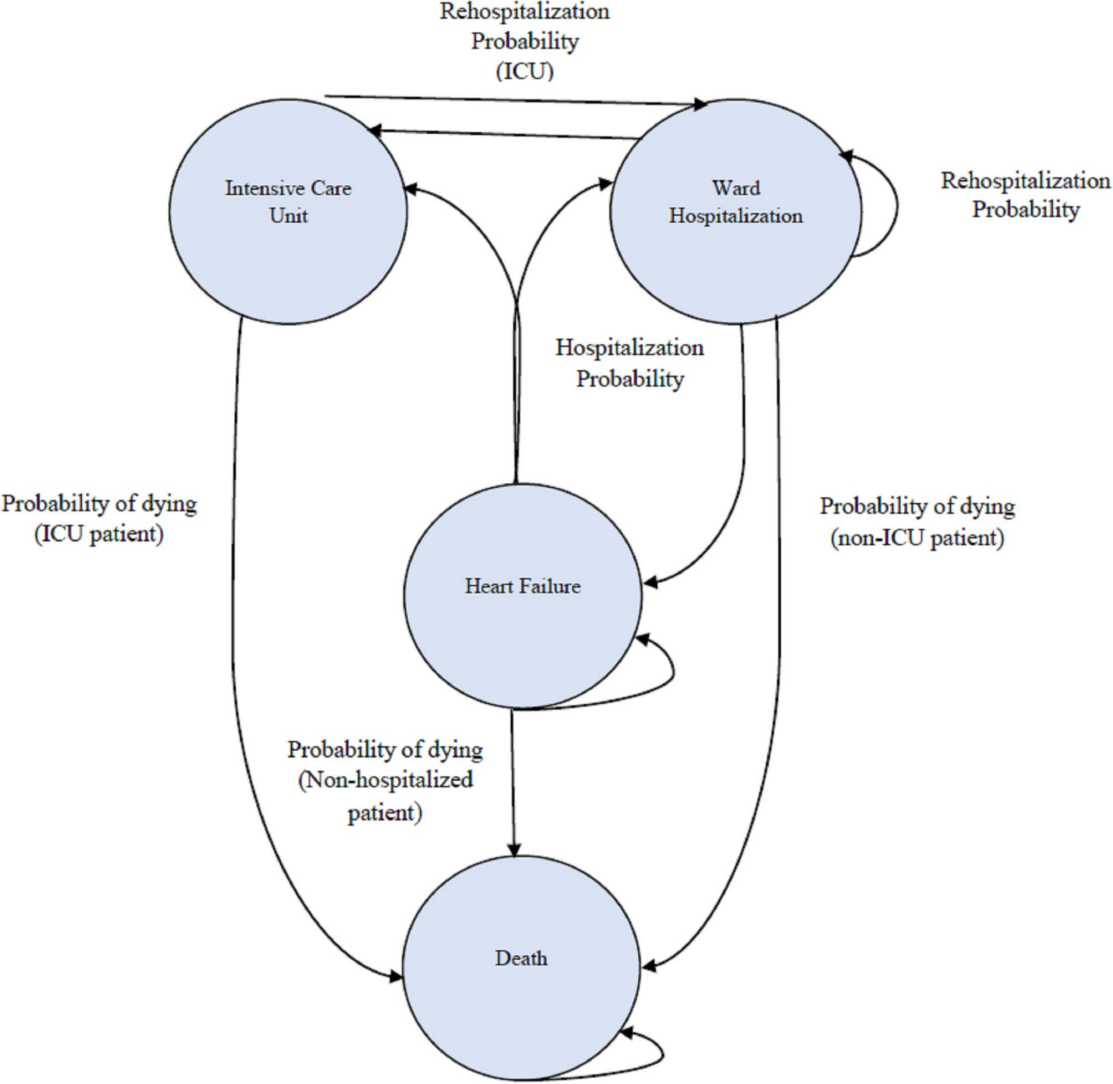
Schematic diagram of Markov model. In this diagram, circles indicate health states; arrows represent all possible transitions between health states. Patients with HF may remain in the same state, be admitted to the general hospital wards (ward hospitalization), be admitted to the intensive care units, or die

In this model, patients were distributed among Markov states with a cycle length of one month based on transition probabilities (the probability of patients passing through different Markov states). These states included outpatients with HF, patients with HF admitted to the general hospital wards, patients with HF admitted to the intensive care units (ICUs), and death [38] (Fig. 1). According to Fig. 1, patients with HF may remain in the same state, be admitted to the general hospital wards (ward hospitalization), be admitted to the intensive care units, or die.

The present study followed the CHEERS (Consolidated Health Economic Evaluation Reporting Standard) checklist [39]. The checklist is available in the appendix.

### Time horizon

Due to the age of onset of the disease of patients in the Markov model (i.e. 24 years) and the Markov states, the time horizon of the lifetime was used, which has also been used in other internal and external studies [40].

### Input parameters

#### Transition probabilities

Due to the lack of related internal and Iranian studies on the transition possibilities, data on the probability of transitions were extracted from international studies, and data related to mortality rate due to other causes were extracted from the age-specific Iranian life Table [41]. Also, the probabilities of hospitalization and death were extracted from PARADIGM-HF [42]. The results of PARADIGM-HF showed that the rates of death and hospital admission in patients who were treated with valsartan, compared to patients treated with enalapril, had decreased by 10% and 21%, respectively.

### Costs

In the current study, the payers’ perspective was used to calculate the costs, and direct medical costs were extracted from internal studies and entered into the Markov model (Table 1). Cost items in the present study included ward hospitalization, ICU, hospitalization for HF per month, and the costs of valsartan, candesartan, and enalapril. Considering that the cycle length was monthly, the costs were also monthly. Given that according to the Markov model, HF patients can be hospitalized in the ward and ICU, the costs of the ICU were also considered for these patients. In the current study, the costs of the Coronary Care Unit (CCU) were considered for ward hospitalization. To estimate the costs of ward hospitalization, ICU, and CCU, the average monthly length of stay for heart failure patients was extracted from the internal study [43] and according to the national tariff Table [44], the costs were calculated.

**Table 1:** Input parameters used in the study economic model

Variable	Transition probabilities used in the Markov model			
	Probability	Standard Error	Distribution	Source
Death (HF)	0.0089	0.001	Beta	[51]
Death (in hospitals)	0.037	0.004	Beta	[52]
Death (in ICUs)	0.11	0.01	Beta	[53]
Ward Hospitalization	0.156	0.05	Beta	[54]
ICU Hospitalization	0.147	0.02	Beta	[54]
Rehospitalization within 30 days	0.199(enalapril/candesartan) 0.088 (valsartan)	0.02 0.006	Beta Beta	[55] [55]
	Effects (risk ratio) of valsartan compares to enalapril (95% CI)			
**Target of effect**	**Risk ratio**		**Distribution**	**Source**
Ward Hospitalization	0.77 (0.67–0.89)	0.06	Log-Normal	[38]
ICU admission	0.82 (0.72–0.94)	0.06	Log-Normal	[38]
Death	0.84 (0.76–0.93)	0.04	Log-Normal	[38]
	Effects (risk ratio) of candesartan (95% CI)			
Ward Hospitalization	0.68 (0.57–0.81)	0.06	Log-Normal	[38]
ICU admission	1			[38]
Mortality	0.87 (0.74–1.03)	0.14	Log-Normal	[38]
	Yearly utility scores for HF patients			
**Model state**	**Utility scores (lower bound- upper bound)**	**Standard Error**	**Distribution**	**Source**
No hospitalization	0.85	0.15	Beta	[40]
Hospitalization	0.828	0.14	Beta	[40]
	The cost items of valsartan, enalapril and candesartan (USD)			
**Cost items**	**Costs (USD)**	**Standard Error**	**Distribution**	**Source**
Ward hospitalization	239	190	Gamma	[43, 44]
ICU	1,112	873	Gamma	[43, 44]
CCU	556	436	Gamma	[43, 44]
The average cost of hospitalization for HF per month	9,312	6,392	Gamma	[43, 44]
Valsartan	6.4	2.56	Gamma	Experts’ opinion
Candesartan	10.2	3.3	Gamma	[38]
Enalapril	4.3	1.02	Gamma	Experts’ opinion

The costs of valsartan and enalapril were obtained by asking the pharmacies’ technical managers (Table 1). Considering that at the time of the study, candesartan was not available in pharmacies, the costs of this medicine were extracted from the van der Pol’s study [38].

The price index and exchange rate in the study year (2020) were taken from the Central Bank website and calculated and reported based on the USD so that each US dollar was considered equivalent to 42,000 Iranian Rials [45].

### Health outcomes

The effectiveness measure in this study was the QALYs. The utility value for each Markov state was extracted from an internal study [40] (Table 1).

### Discounting

Considering that the time horizon in the present study was the lifetime, discount rates of 5.8% and 3% were applied for costs and QALYs, respectively [46, 47].

### Willingness to pay

In the present study, the threshold value was calculated based on the method proposed by the World Health Organization (WHO), i.e. between one and three times the GDP per capita, and the former was used in this study (7,256 USD in 2020) [48].

### Sensitivity analyses

In the next step, the ICER was calculated by the following formula [49]

$$\text{I}\text{C}\text{E}\text{R}=\frac{\text{C}\text{o}\text{s}\text{t}\text{s}\, \text{o}\text{f}\, \text{v}\text{a}\text{l}\text{s}\text{a}\text{r}\text{t}\text{a}\text{n}-\text{C}\text{o}\text{s}\text{t}\text{s}\, \text{o}\text{f}\, \text{e}\text{n}\text{a}\text{l}\text{a}\text{p}\text{r}\text{i}\text{l}}{\text{Q}\text{A}\text{L}\text{Y}\text{s}\, \text{o}\text{f}\, \text{v}\text{a}\text{l}\text{s}\text{a}\text{r}\text{t}\text{a}\text{n}-\text{Q}\text{A}\text{L}\text{Y}\text{s}\, \text{o}\text{f}\, \text{e}\text{n}\text{a}\text{l}\text{a}\text{p}\text{r}\text{i}\text{l}}$$

In economic evaluation studies, uncertainty is an inevitable factor. Therefore, in this study, the robustness of the results was examined using the one-way and probabilistic sensitivity analyses. The one-way sensitivity analysis (by drawing the Tornado diagram) and the probabilistic analysis were used to determine the robustness of the results. The probabilistic sensitivity analysis diagram was drawn using the Monte Carlo simulation, assigning the Gamma distribution for costs, the Log-Normal distribution for risk ratios, and the Beta distribution for the parameters of utility and transition probabilities. The cost-effectiveness acceptability curve, which is one of the curves helping health system policymakers and planners determine the probability of any intervention cost-effectiveness at different willingness to pay, was also drawn [50]. The TreeAge Pro 2011 software was used for data analysis.

## Results

### Costs

The total mean costs for valsartan, candesartan, and enalapril were 62.4 USD, 66.2 USD, and 60.3 USD, respectively.

### QALYs

The utility values for No hospitalization and Hospitalization were 0.85 and 0.828, respectively, and with regard to the one-month period studied, their QALYs were, respectively, 0.071 and 0.069.

### ICER

The results showed that the average expected costs and QALYs were 119,645.45 USD and 16.15 for valsartan, 113,019.68 USD and 15.16 for enalapril, and 113,093.37 USD and 15.06 for candesartan, respectively.

**Fig. 2:**
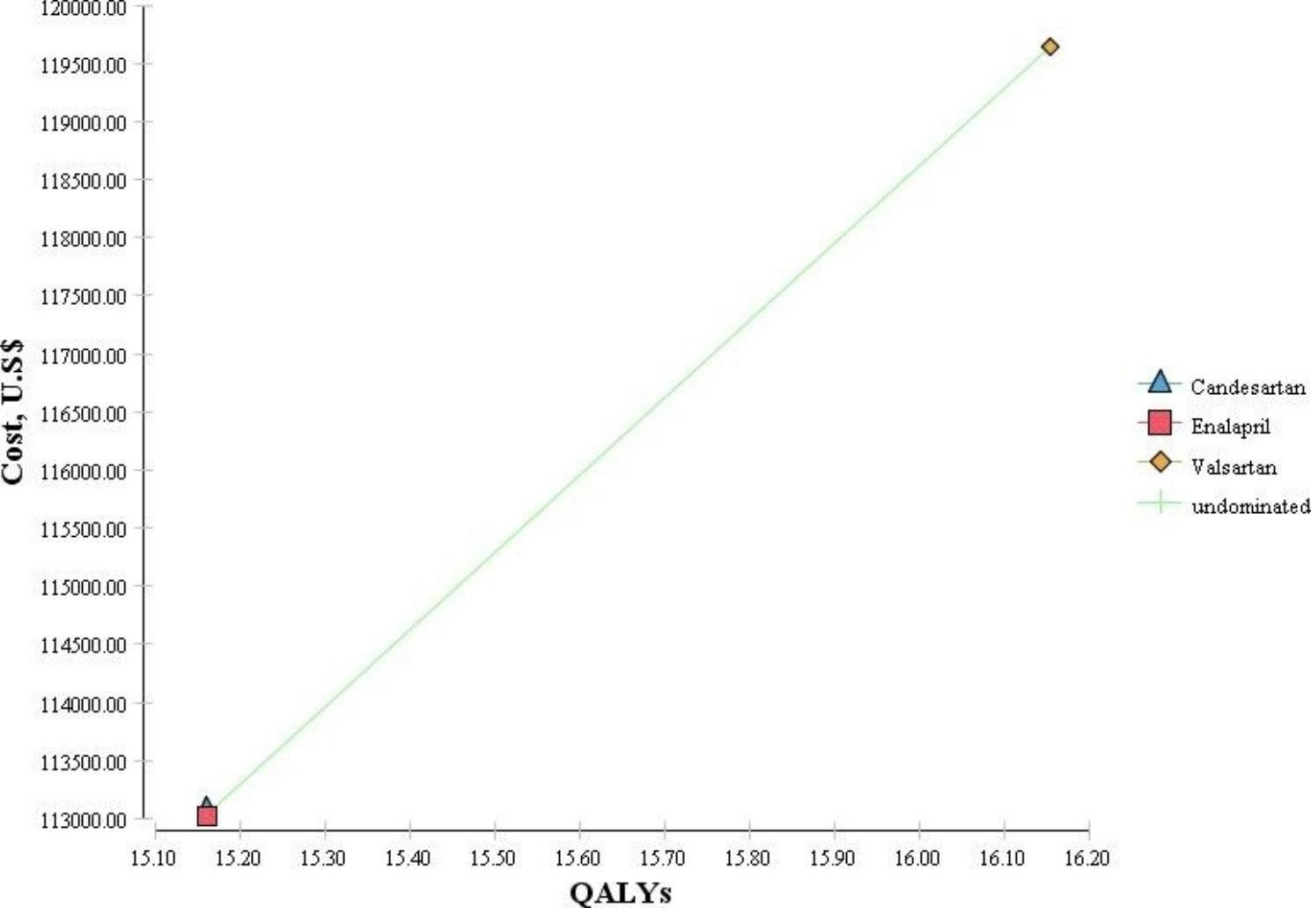
The results of the cost-effectiveness analysis

According to Table 2 and Fig. 2, candesartan was known as the dominated option due to its highest cost and lowest QALYs among the three studied medicines. However, valsartan had higher costs and QALYs than enalapril. Therefore, to make a decision, the ICER had to be calculated and compared with the threshold value. The results showed that because the calculated ICER value (6,692.69 USD) was less than the threshold value (7,256 USD), valsartan was cost-effective compared to enalapril.

**Table 2:** Base case cost-effectiveness results estimated over the lifetime, discounted

Strategies	Costs (USD)	QALYs	∆C	∆QALYs	Incremental Cost per QALY Gained (valsartan vs. enalapril)
Valsartan	119,645.45	16.15	6,625.77	0.99	6,692.69
Enalapril	113,019.68	15.16			
Candesartan	113,093.37	15.06	Dominated		

QALYs = Quality-adjusted life years; ∆C = cost difference; ∆QALY = QALY difference

### Sensitivity analyses

**Fig. 3:**
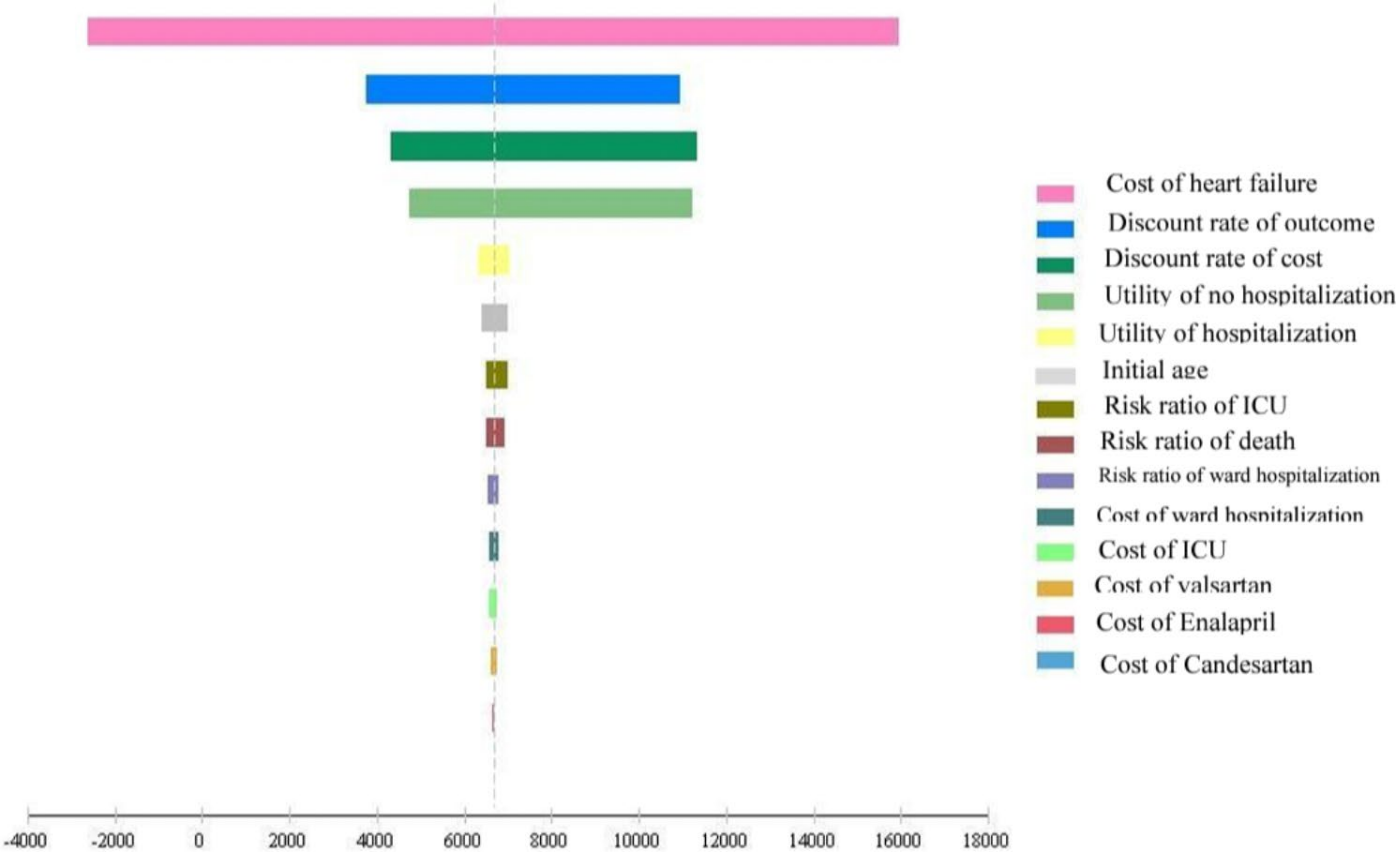
The Tornado diagram of the one-way sensitivity analysis

In the present study, the 95% confidence interval was used for the one-way sensitivity analysis, and then the Tornado diagram was drawn (Fig. 3). The results of the Tornado diagram showed that the results of the study were highly sensitive to the cost of HF, the discount rate of the outcome, the discount rate of cost, and the utility of no hospitalization.

**Fig. 4:**
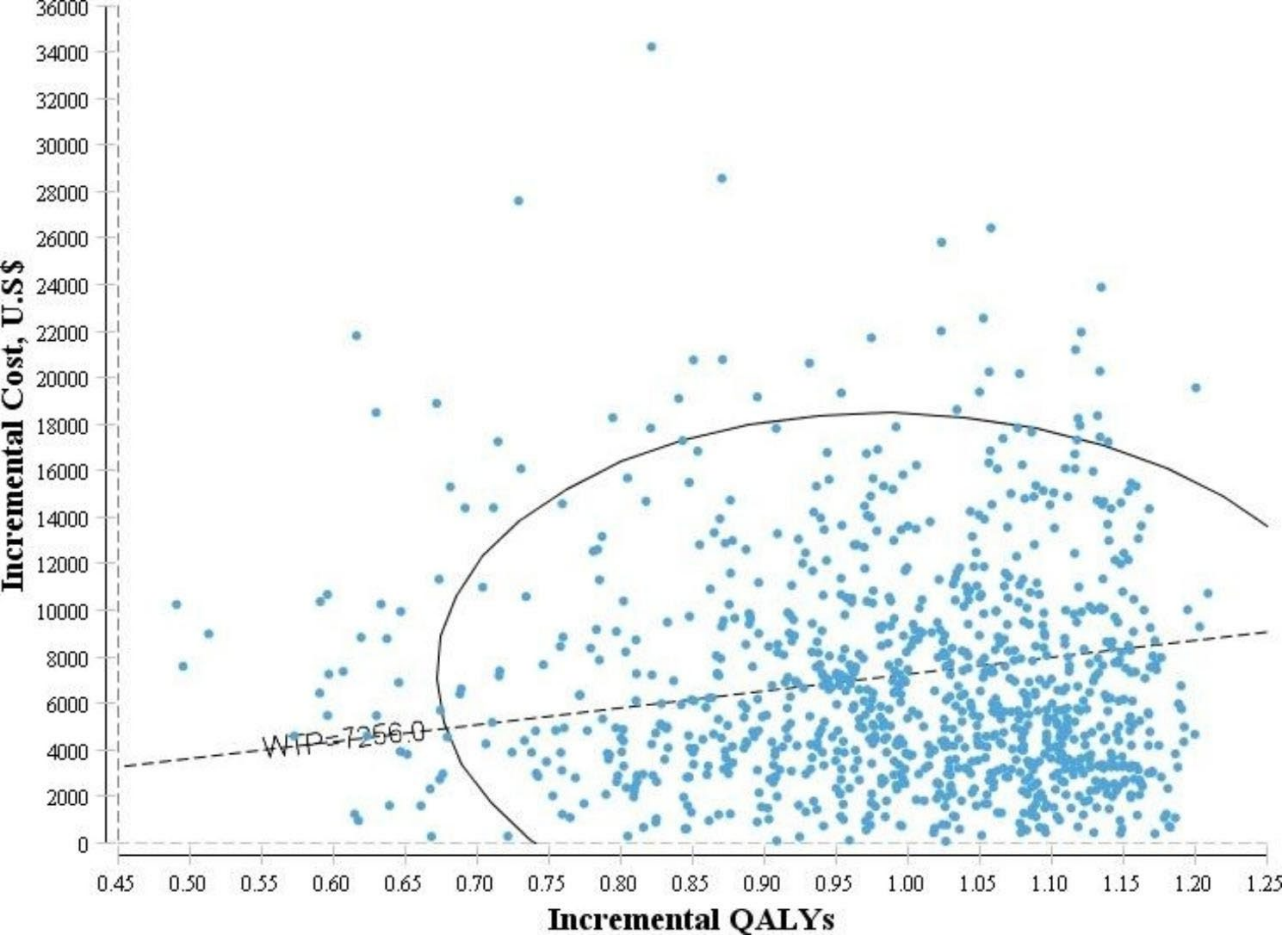
The results of the probabilistic sensitivity analysis

In the probabilistic sensitivity analysis diagram (Fig. 4), the horizontal axis shows the difference in the amount of QALYs and the vertical axis shows the difference in the costs between the two medicines. The results of this diagram showed that the distribution of points was mostly in the first quarter of the cost-effectiveness plane.

**Fig. 5:**
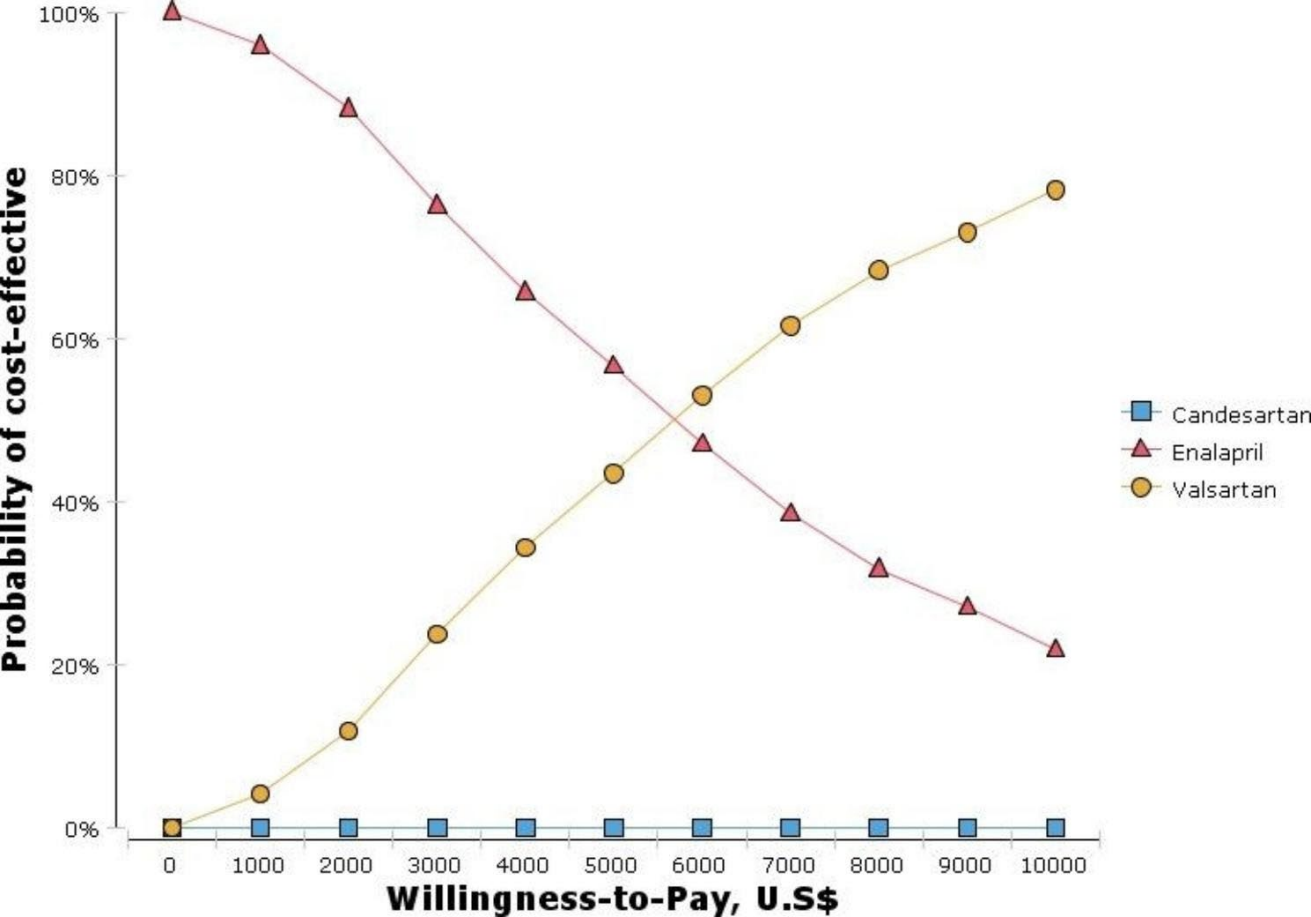
The cost-effectiveness acceptability curve

Figure 5 shows the cost-effectiveness acceptability curve. The results showed that at the threshold of 7,256 USD, valsartan had a 60% chance of being cost-effective compared to enalapril.

## Discussion

HF is one of the leading causes of morbidity, mortality, and readmission to the hospital. The economic burden of the disease has increased dramatically over the past two decades, leading to the waste of resources and increases in the costs of health care systems [56]. In the last three decades, pharmaceutical treatments based on current clinical guidelines, including the use of Angiotensin Receptor Blockers, have shown significant success in reducing hospitalization, morbidity, and mortality associated with HF [57]. On the other hand, economic evaluation studies play an important role in the optimal allocation of resources and appropriate decision-making in health systems, and cost-utility studies are usually considered the gold standard for economic evaluations [58]. This study aimed to compare the cost-utility of candesartan, enalapril, and valsartan in patients with HF using the Markov model in Iran in 2020.

The results of the present study showed that valsartan was cost-effective compared to two other studied medicines. Moreover, the results of the current study showed that ICU had the highest cost among different cost items, which is in line with those of the Jabbari et al. in Iran (2020) and Van der Pol et al. in the Netherlands (2017) [37, 59].

Moreover, the results of the current study showed that the amounts of QALYs for valsartan were estimated to be 16.15, and 15.16 and 15.06 for enalapril and candesartan. The results of this study are in line with those of the studies by Gaziano et al. (2020) in the United States [60], Margarida Borges et al. (2020) in Portugal [61], Van der Pol et al. in Germany (2019) [38], Pradelli et al. (2009) in Italy [62], McMurray et al. (2018) in the three countries of the UK, Denmark and Colombia [63], and the Ademi et al. (2017) in Switzerland [34]. In their study, King et al. (2016) found that valsartan had higher cost and effectiveness for patients with HF than enalapril and that the valsartan’s higher cost-effectiveness depended on the patients’ duration of treatment [64]. However, the results of the Krittayaphong and Permsuwan’s study (2018) showed that valsartan, compared to enalapril, did not represent good value because of its high price in Thailand [32]. Liang et al. (2018) also concluded in their study that valsartan did not represent good value, compared to enalapril, in reducing morbidity and mortality in patients with HF in Singapore [65]. The results of these two studies are not in line with those of the present study.

In the current study, sensitivity analyses were performed to show the robustness of the results relative to the model parameters and the results of the Tornado diagram showed that the results of the study were highly sensitive to the cost of HF, the discount rate of the outcome, the discount rate of cost, and the utility of no hospitalization. Moreover, the results of the cost-effectiveness acceptability curve showed at the threshold of 7,256 USD, valsartan had a 60% chance of being cost-effective compared to enalapril. and with an increase in willingness to pay to 10,000$, the probability of being cost-effective increases to 85%.

Given that valsartan is easily available at a reasonable price in Iran, it can be a good treatment option for patients with HF in this country. However, because of differences in cost coverage by health insurance organizations, differences in the effectiveness of diagnostic tests such as echocardiography and exercise testing, patients’ willingness to pay, and different prevalence of HF in different countries, it is necessary to be cautious in generalizing the results of the present study to other countries.

The strengths of the present study were the use of the lifetime time horizon, the use of the Markov model that was validated and used in previous studies, and the extraction of utility and cost values of the Markov states from internal studies.

However, the current study, like other studies, had some limitations. One of the limitations of the present study was to use cohort simulation and for future study, it is suggested the researchers apply other simulation techniques such as system dynamics, discrete event simulation, and agent-based simulation.

Due to the lack of internal and Iranian studies on the transition probabilities, the required data were extracted from external studies. Another study limitation was that the payers’ perspective did not take into account the patients’ direct non-medical and indirect costs, and therefore it is suggested to use the societal perspective that examines all types of costs in future studies. It should be noted that in the present study, the costs extracted from other studies were updated and adjusted according to their study time, and the effect of changes in costs and utility values ​​was investigated using sensitivity analyses. Overall, it is very necessary to conduct economic evaluation studies on medicines in low- and middle-income countries due to their severe limitations of health resources and the need to make optimal use of the resources available. To evaluate and determine the cost-effectiveness of new medicines in such countries and to provide guidance in decision-making, it is suggested that more economic evaluation studies, such as the present study, be conducted in these countries in various fields.

Although medicines such as telmisartan, azilsartan, olmesartan, and irbesartan are alternative medicines to valsartan, because these medicines were not included in the Iran pharmacopeia, they were not investigated in the current study and it is suggested that in future studies in countries where these medicines are part of their pharmacopeia, they should also be studied and compared.

## Conclusion

According to the results of the present study, valsartan was cost-effective compared to enalapril and candesartan and it is recommended that when designing clinical guidelines for disease control of patients with HF, health policymakers consider the use of valsartan by cardiologists. In this study, we used cohort simulation and for future study, it is suggested the researchers apply other simulation techniques such as system dynamics, discrete event simulation, and agent-based simulation.

### Electronic supplementary material

Below is the link to the electronic supplementary material.

Additional file 1: CHEERS Checklist

Additional file 2: Total cost and QALYs for different health states

## Data Availability

All related data were displayed in the manuscript. Further information regarding the data can be obtained by contacting the corresponding authors.
