# Supplementary material for: Cost-utility analysis of valsartan, enalapril, and candesartan in patients with heart failure in Iran
Source: Health Econ Rev. 2023 Sep 4;13:44. doi: 10.1186/s13561-023-00457-4 (PMC10476319; doi:10.1186/s13561-023-00457-4)
Supplement: Supplementary file 2 — Additional file 2: Total cost and QALYs for different health states [file 13561_2023_457_MOESM2_ESM.docx]

| Health states | Total cost (USD) | QALY |
| --- | --- | --- |
| CCU | 6672 | 27.2 |
| Ward hospitalization | 2868 | 28.4 |
| ICU | 13344 | 25.7 |

**Additional file 2. Total cost and QALYs for different health states**
